# Cholinergic modulation is independent of T lymphocytes in a mouse model of neuropathic pain

**DOI:** 10.1177/17448069221076634

**Published:** 2022-02-17

**Authors:** Katherine Halievski, Ameet S Sengar, Janice Hicks, Jillian Haight, Michael W Salter, Benjamin E Steinberg

**Affiliations:** 1 7979 The Hospital for Sick Children, Toronto, ON, Canada; 2 7989 University Health Network, Toronto, ON, Canada

**Keywords:** Neuropathic pain, animal models, T lymphocytes, cholinergic signaling, choline acetyltransferase

## Abstract

T lymphocytes are increasingly implicated in pain signaling. A subset of T lymphocytes, termed TChAT, express the rate-limiting enzyme for acetylcholine (ACh) production, choline acetyltransferase (ChAT), and mediate numerous physiological functions. Given that cholinergic signaling has long been known to modulate pain processing and is the basis for several analgesics used clinically, we asked whether TChAT could be the intersection between T lymphocyte and cholinergic mediation of pain signaling. In this study, we used a mouse gene knockout strategy to ablate ChAT specifically from T lymphocytes and examined the development and expression of mechanical and thermal hypersensitivity in a spared nerve injury (SNI) mouse model of neuropathic pain. We found that mice with ChAT knockout in T cells (floxed *Chat* plus CD4-Cre recombinase) did not differ from control mice with intact ChAT (floxed *Chat*, but no Cre recombinase) in their expression of mechanical sensitivity before or after injury. Similarly, thermal sensitivity was unaffected after injury, with control mice expressing similar patterns of thermal preference to mice whose T cells do not express ChAT. Our experiments demonstrate that cholinergic signaling initiated by T lymphocytes neither dampens nor exacerbates the expression of mechanical or thermal sensitivity in neuropathic mice. Thus, while both cholinergic signaling and T lymphocytes have established roles in modulating pain phenotypes, it is not cholinergic signaling initiated by T lymphocytes that drive this. Our findings will help to narrow in on which aspects of T-cell modulation may prove useful as therapies.

## Introduction

The cholinergic system plays an important role in neuropathic pain processing.^1^ To date, research on pain modulation by acetylcholine (ACh) has focused on central pathways at both the spinal and supraspinal levels, and acetylcholinesterase inhibitors, which increase ACh levels around the spinal cord, are used clinically in epidural analgesia for postoperative and labor-induced pain patients.^2^ The role of the peripheral cholinergic system, on the other hand, is not as well defined, though it may be a major contributor to neuropathic pain.^1^ Indeed, nicotinic acetylcholine receptors (AChRs) are expressed in mammalian peripheral sensory neurons at primary afferent terminals^3^ and in non-neural cells, including immune cells.^4^ One immune cell type that has been increasingly implicated in pain signaling is the T cell, which can drive both nociception^5^ and antinociception,^6^ possibly in age-^7^ and sex-dependent^8^ manners. A specialized subset of T lymphocytes express choline acetyltransferase (ChAT), the rate-limiting enzyme necessary for ACh production, and synthesize and release this neurotransmitter, which can act on peripheral AChRs.^9^

We and others have shown that these ChAT-expressing lymphocytes, termed TChAT, regulate important physiological processes—hemodynamic responses, anti-viral pathways, and immune-modulating neural circuits.^9–11^ As such, we hypothesized that TChAT may be the interface between nervous and immune systems—and possibly the main mediator of cholinergic pain signaling in the periphery—through their release of ACh, which modulates AChR activation. If TChAT are found to be involved in the cholinergic mechanisms of pain, they could potentially serve as a target for modulating the manifestation of neuropathic pain. In this study, using a cell-specific knockout of ChAT, we evaluated the role of this enzyme in T lymphocytes in the nociceptive behavior of a model of neuropathic pain that is known to involve T cells.^7,8^

## Methods

### Animals

Experiments were approved by the Hospital for Sick Children Animal Care Committee and conducted in accordance with animal care regulation and policies of the Canadian Council on Animal Care. *Chat*^flox/flox^ (B6.129- ChAT^tm1Jrs^/J) and CD4-Cre (Tg (Cd4-cre)1Cwi/BfluJ) mice were housed in same-sex polycarbonate cages with *ad libitum* access to food and water. Housing rooms were temperature and humidity controlled with 14:10 h light:dark cycles. All examined animals were analyzed and investigators were blinded to genotype.

### Surgical model

The spared nerve injury model (SNI) model of neuropathic pain was used.^12,13^ Briefly, under isoflurane anesthesia, the biceps femoris muscle was bluntly dissected, exposing the sciatic nerve. The common peroneal and tibial nerves were ligated and cut, leaving the sural nerve intact. Control animals underwent sham surgery, which included all elements of the SNI surgery except nerve ligation and transection.

### Behavioral assays

For behavioral testing by von Frey algesiometry, mice were placed in plastic cubicles on a perforated metal platform (Ugo Basile) and habituated for 60 min. Calibrated filaments (Touch Test Sensory Evaluator, Stoelting; 0.02–1.4 g) were applied to the lateral portion of the plantar hindpaw. The up-down method of Dixon was used to determine the 50% mechanical withdrawal threshold.^14^ Each data point was averaged from two trials on one day, except for baseline which was the average from two days.

Thermal sensitivities were measured using the Thermal Place Preference apparatus (Bioseb). The protocol included a total of 24.5 min of simultaneous temperature ramping on two plates (Plate 1: 7 min at 25°C, 10 min transition from 25°C to 45°C, and a 7.5 min transition from 45°C to 30°C; Plate 2: 5 min at 25°C, 2 min transition from 25°C to 45°C, and a 17.5 min transition from 45°C to 10°C). Mouse position was detected and quantified automatically by Bioseb software with data acquired by an overhead camera.

### Intrathecal injection

Neostigmine (catalog no: N2001; Sigma-Aldrich Canada) was administered intrathecally to non-anesthetized mice with a 30G needle at a dose of 6.25 μg in 5 μL saline.

### Statistical analysis

Data are reported as mean ± SEM, with *n* indicating the sample size, and analyzed using SPSS Statistics version 26 and GraphPad Prism version 9. Statistical tests are reported in the figure legends. Values of *p* < .05, corrected for multiple comparisons, were considered statistically significant.

## Results

To evaluate the role of ChAT-expressing T lymphocytes in neuropathic pain, we used a Cre-lox recombination strategy in mice to genetically delete ChAT in CD4^+^ lymphocytes. *Chat*^flox/flox^ mice were crossed with mice expressing Cre recombinase under the control of the endogenous CD4 promoter to generate *Chat*^flox/flox^ CD4-Cre^−^ wildtype (TChAT^WT^) and *Chat*^flox/flox^ CD4-Cre^+^ (TChAT^KO^) knockout mice.

**Figure 1.**
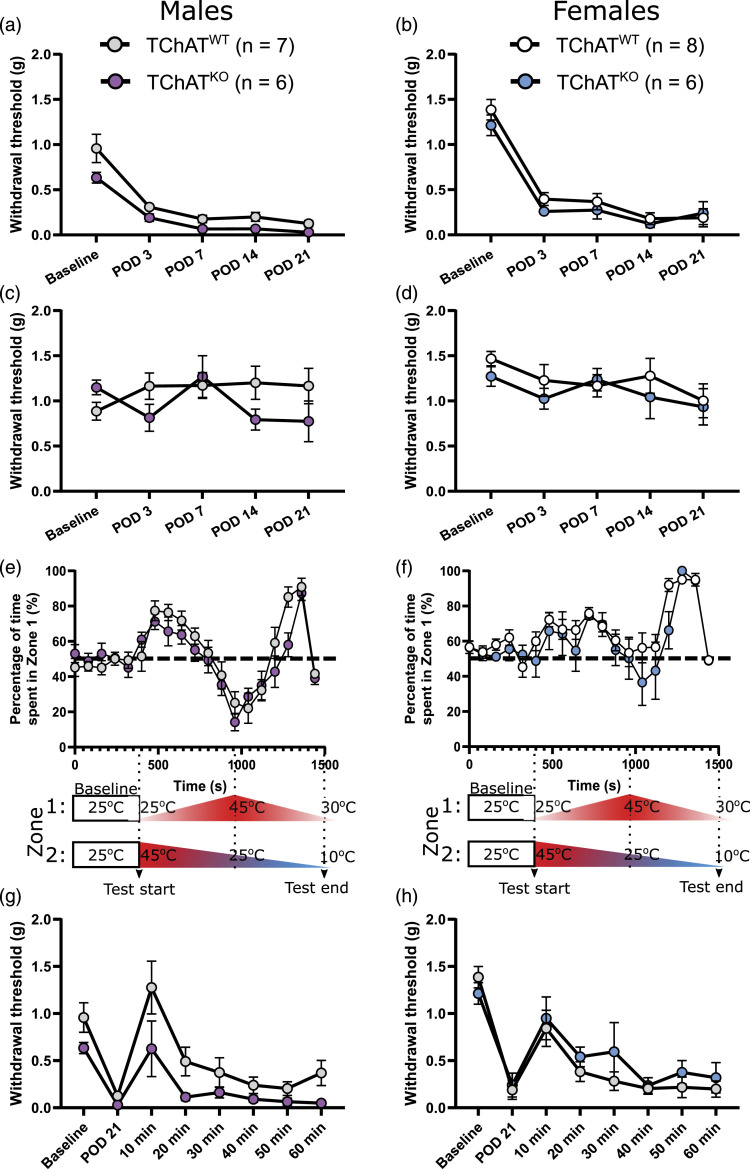
Ablation of choline acetyltransferase (ChAT) from T lymphocytes does not impact the expression of mechanical or thermal sensitivity in a mouse model of neuropathic pain**.** a, b) Mechanical sensitivity was measured using von Frey algesiometry in (a) male and (b) female mice before (baseline) and after spared nerve injury (postoperative day [POD] 3, 7, 14, and 21). Both male and female mice expressing ChAT in T cells (ChAT^WT^) and mice with T-cell ChAT knockout (TChAT^KO^) showed statistically similar levels of sensitivity at all timepoints tested (no significant group differences for males and females), though they did develop hypersensitivity across time following SNI (*p* < 0.001 for males and females). c, d) The paw contralateral to injury was also tested with von Frey filaments, and no differences were observed between ChAT^WT^ and TChAT^KO^ groups in either sex, nor were differences observed across time after injury. e, f) Thermal (hot and cold) sensitivity was measured 2 weeks after SNI in (e) males (POD18) and (f) females (POD16) using a thermal place preference apparatus, where the temperatures of two thermal plates changed across time. Data represent the percent time spent on Plate 1 (Zone 1) by each group of mice, where 50 percent—as shown by the dashed line—indicates no preference. Each thermal place preference data point was acquired in an 80-s bin and is reported as a percentage. Plate 1 (Zone 1) and Plate 2 (Zone 2) each started with a 5-min habituation period at 25°C, and then shifted temperatures as indicated underneath panels e and f. While there was a main effect of temperature (i.e., a thermal preference; *p* < 0.001) in both male and female ChAT^WT^ and TChAT^KO^ mice, there was no interaction between temperature and genotype, indicating that knockout of ChAT does not impact thermal sensitivity in mice that have undergone a nerve injury. g, h) Analgesic effects of intrathecal neostigmine, a ChAT inhibitor, are equally effective in mice with and without ChAT expression in T lymphocytes. In both males (g) and females (h), intrathecally administered neostigmine (6.25 μg in 5 μL saline in non-anesthetized mice) resulted in a transient reversal of mechanical hypersensitivities 10 min after treatment, regardless of the genotype. Mechanical sensitivity, as measured with von Frey filaments, reached pre-surgery (baseline) levels at the peak of neostigmine’s short-lasting effect. This was observed across all groups (baseline versus 10 min, *p* > 0.05 in each of the 4 groups), indicating that neostigmine was not differentially effective in one group over another. This indicates that cholinergic analgesia exists even in mice whose T lymphocytes cannot produce ACh. Male mice (ChAT^WT^: *n* = 7, age range at POD0 = 207–235 days; TChAT^KO^: *n* = 6, age range at POD0 = 207–216 days) and female mice (ChAT^WT^: *n* = 8, age at POD0 = 215 days; TChAT^KO^: *n* = 6, age at POD0 = 215 days). Mann–Whitney *U* with a Bonferroni correction for multiple comparisons was used to analyze group differences in panels a–d, and a mixed design repeated measures ANOVA was used to analyze data in panels (E) and (F), with genotype as the independent variable and temperature as the repeated measures variable. A Friedman test was used to test for differences across time in panels A–D. A Wilcoxon matched-pairs signed rank test was used to test for differences across time in panels (G) and (H). Baseline and POD21 data are the same as reported in panels (A) and (B), and are included in panels (G) and (H) for reference.

We conducted experiments in males and females, since sex differences exist in the involvement of T cells in pain.^8^ We first tested baseline sensitivities prior to SNI and found that mechanical sensitivity did not differ between TChAT^WT^ and TChAT^KO^ mice in males and females (leftmost datapoints in Figure 1(a)–(d)). We next evaluated nociceptive behavior in the SNI model, which exhibits prominent mechanical hypersensitivity. Following injury, all groups showed mechanical hypersensitivity in the ipsilateral hindpaw as early as day 3 after surgery, which persisted for at least 21 days (Figure 1(a) and (b); POD3, POD7, POD14, POD21). Following induction of SNI, we also tracked mechanical thresholds in the contralateral hindpaw and found no mechanical hypersensitivity in any of the groups (Figure 1(c) and (d)).

As pain modalities are regulated by different stimuli, neural circuits, and signaling pathways, and are differentially regulated following injury, we further tested thermal sensitivities following SNI. We tested both cold and hot sensitivities using the thermal place preference test, and discovered that this, too, did not differ between genotypes in either sex (Figure 1(e) and (f)). Our data demonstrate that neither thermal nor mechanical hypersensitivities are affected by a loss of ChAT from T lymphocytes, allowing us to exclude this cell type as a candidate source of ACh in painful contexts.

## Discussion

Both T lymphocytes and cholinergic signaling have established roles in mediating chronic pain, yet their intersection has never been assessed. In the present study, we genetically ablated TChAT and found that their absence does not alter either basal sensitivities or the ontogeny of neuropathic pain–like behavior in male or female mice, strong evidence that T lymphocytes do not mediate cholinergic signaling as it relates to pain.

Eliminating T lymphocytes globally (e.g., CD4-KO, Rag1-KO, or *nude* mice) modulates neuropathic pain,^5,6,8^ and our data show that it is *not* the cholinergic functions of T cells that mediate their pain-modulating effects in mice. In fact, we also tested cholinergic analgesia using neostigmine (a ChAT inhibitor that enhances extracellular levels of ACh)^15^ in male and female TChAT^KO^ and TChAT^WT^ mice and found that it induced a transient reversal of mechanical hypersensitivity in both sexes and genotypes (Figure 1(g) and (h)), indicating that cholinergic analgesia exists even in mice whose T lymphocytes cannot produce ACh. If not T cells, what could be the source of ACh conferring analgesia? ChAT, marking cholinergic cells, is expressed by inhibitory interneurons of the spinal dorsal horn^16^ and by primary afferents of the dorsal root ganglion.^17,18^ It is also possible that ACh-induced analgesia is derived from other immune cells, such as B lymphocytes, dendritic cells, or macrophages, as these cells can express *Chat* mRNA.^19^ The identification of the cellular sources of ACh-induced analgesia remains an active area of research.

Together, our findings indicate that T cell–mediated pain and cholinergic analgesia do not occur via the same mechanism. While the role of T lymphocytes in pain is pronounced, it is also nuanced: depending on the context, T cells may be nociceptive or antinociceptive.^5,6^ Thus, questions regarding how T cells mediate pain remain—what T-cell subtypes, where do they act, via what signaling pathways, and in which subjects? Such studies will clarify how T cell–targeted therapies can be leveraged to treat chronic pain.
